# The tumor-suppressive function of miR-1296-5p by targeting EGFR and CDK6 in gastric cancer

**DOI:** 10.1042/BSR20181556

**Published:** 2019-01-08

**Authors:** Yan Jia, Lian-Mei Zhao, Han-Yu Bai, Cong Zhang, Su-Li Dai, Hui-lai Lv, Bao-En Shan

**Affiliations:** 1 Research Center, Fourth Hospital of Hebei Medical University, Shijiazhuang, Hebei Province, 050011, China; 2 Department of Cardiology, The Second Hospital of Hebei Medical University, Shijiazhuang, Hebei Province, China; 3 Department of Thoracic Surgery, Fourth Hospital of Hebei Medical University, Shijiazhuang, Hebei Province, 050011, China

**Keywords:** CDK6, EGFR, Gastric Cancer, miR-1296-5p

## Abstract

We aimed to confirm the role of miR-1296-5p in gastric cancer and to identify its target genes. The expression of miR-1296-5p was measured in gastric cancer tissues and cell lines. The function of miR-1296-5p was examined by the overexpression and inhibition of its expression in typical gastric cell lines as well as SGC-7901 and MGC-803 cells. The targets of miR-1296-5p were identified by a luciferase activity assay. We found that miR-1296-5p was down-regulated in gastric cancer tissue and cell lines, and low expression levels of miR-1296-5p were associated with advanced clinical stage. Moreover, miR-1296-5p inhibited cell proliferation, migration, and invasion in SGC-7901 and MGC-803 cells. Then, we identified CDK6 and EGFR as novel targets of miR-1296-5p by a luciferase activity assay. Furthermore, the overexpression of miR-1296-5p suppressed the expression of CDK6 and EGFR. Our results indicated a tumor-suppressive role of miR-1296-5p through the translational repression of oncogenic CDK6 and EGFR in gastric cancer.

## Introduction

Gastric cancer (GC) is the fifth most common cancer after cancers of the lung, breast, colorectum, and prostate, and it is the third most common cause of cancer-related death worldwide [1,2]. The clinical outcomes of patients affected by GC are still not encouraging; indeed, the 5-year survival rate is less than 30% [3,4]. The development and progression of GC are thought to be a multistep process involving the accumulation of several genetic mutations and alterations in proto-oncogenes or tumor-suppressor genes. Unfortunately, most patients with GC have poor prognosis because this cancer is typically diagnosed at a late stage of disease [5–7]. Therefore, further insight into the molecular mechanisms underlying GC progression may identify novel therapeutic targets and improve the prognosis of GC.

MicroRNAs (miRNAs) are a subset of small non-coding RNA molecules that are believed to regulate gene expression by binding to partially complementary sequences of target mRNAs [8,9]. Emerging evidence has revealed that miRNAs are related to several aspects of cancer pathogenesis, including self-renewal, invasion, and metastasis, by targeting cyclin-dependent kinase 6 (CDK6), epidermal growth factor receptor (EGFR), MAPK, and PI3K/AKT signaling pathways [10–14]. miR-1296-5p, a novel cancer-related miRNA, has been found to be dysregulated in various cancers. miR-1296-5p acts as a tumor suppressor in reproductive and digestive system tumors, including prostate cancers, cervical cancer, breast cancer, hepatocellular carcinoma, and GC [15–19]. However, the target genes of miR-1296-5p may change in different contexts. miR-1296 inhibits the expression of minichromosome maintenance protein 2 (MCM2) in prostate cancer. Xu et al. [19] showed that miR-1296 inhibits the migration, invasion, and EMT progression of hepatocellular carcinoma by the suppression of SRPK1 and downstream PI3K/AKT signaling. Phan et al. [17] identified cyclin D1 as a target of miR-1296 in triple-negative breast cancer cell lines. Shan et al. [18] found that miR-1296-5p suppressed the migration and invasion of human GC cells in part by targeting the ERBB2/Rac1 signaling pathway. As shown by these previous studies, miR-1296-5p may play a tumor-suppressive role by influencing the gene expression of several targets.

CDK6, in cooperation with cyclin D, propels cell cycle progression from the G1 to the S phase through the phosphorylation and subsequent inactivation of the retinoblastoma 1 (RB1) protein. The CDK6 gene is frequently amplified or overexpressed in a variety of human tumors [12].

The EGFR gene is located at the chromosomal region 7p12 and encodes a 70 kDa transmembrane tyrosine kinase receptor that contributes to cancer progression by mediating cellular proliferation, migration, adhesion, and metastasis [20]. Approximately 30% of GC patients have been reported to show EGFR protein overexpression, and thus, EGFR signaling pathways serve as attractive therapeutic targets [21,22].

The purpose of the present study was to confirm the role of miR-1296-5p in GC and to identify the target gene of miR-1296-5p in GC.

## Materials and methods

### Clinical samples and cell lines

GC tissues and matched adjacent non-tumor normal tissues were obtained from 40 patients undergoing radical gastrostomies at the Department of Thoracic Surgery of the Forth Hospital of Hebei Medical University, from 2016 to 2017. None of the patients received preoperative treatment, chemotherapy, or radiotherapy. Ethical approval of the study was granted by the Clinical Research Ethics Committee at Hebei Medical University. Written informed consent was obtained from each participant. The samples were immediately placed in TRIzol reagent (Invitrogen, Carlsbad, CA), homogenized, and stored at −80°C prior to analysis by quantitative real-time PCR or prior to being fixed in formalin for analysis by immunohistochemistry. All clinicopathological information was recorded. The histological type was defined based on Lauren’s classification. The tumor-node-metastasis (TNM) classification definitions were performed according to the 7th edition of the AJCC (American Joint Committee on Cancer) TNM staging system.

Human GC cell lines SGC-7901, NCI-N87, BGC-823, MGC-803, MKN-28, AGS, and the immortalized gastric epithelial cell line (GES-1) were purchased from the Cellular Biology Institute of the Shanghai Academy of Sciences (Shanghai, China). These gastric cell lines were cultured in RPMI-1640 supplemented with 10% fetal bovine serum (FBS, Gibco) at 37°C in a humidified atmosphere of a 5% CO_2_ incubator.

### RNA extraction and real-time polymerase chain reaction

miRNA from gastric tissues and cells was isolated with TRIzol reagent (Invitrogen, Carlsbad, CA) and further purified using a mirVana™ miRNA isolation kit (Ambion, Austin, TX) according to the manufacturer’s instructions. RNA concentrations were measured with a Nanodrop ND-1000 spectrophotometer (Nanodrop Technologies, Wilmington, DE, U.S.A.). RNA samples were reverse-transcribed to complementary DNA (cDNA) using random hexamers and MMLV reverse transcriptase (Invitrogen). Quantitative real-time PCR was performed using a GoTaq PCR Master Mix kit (Promega) on a GeneAmp7300 RT-PCR System (Applied Biosystems, MS). The expression of miR-1296-5p was normalized to U6 expression. RNA expression was calculated with the 2^−ΔΔCt^ method. The primers were designed as follows: miR-1296-5p RT primer 5′- GTC GTA TCC AGT GCG TGT CGT GGA GTC GGC AAT TGC ACT GGA TAC GAC GGA GAT-3′, miR-1296-5p PCR primer 5′-GTTAGGGCCCTGGCTCC-3′, miR-1296-5p universal primer 5′-CAG TGC GTG TCG TGG AGT-3′, U6 forward primer 5′-GCT TCG GCA GCA CAT ATA CTA AAA T-3′, U6 reverse primer 5′-CGC TTC ACG AAT TTG CGT GTC AT-3′, U6 RT primer 5′-CGC TTC ACG AAT TTG CGT GTC AT-3′, EGFR forward primer 5′-ACG CAG TTG GGC ACT TTT GAA-3′, EGFR reverse primer 5′-CAT GTG AGA CTT TGA GTA GAC CTG A-3′, CDK6 forward primer 5′-AGA GCA AGA TAA TAA AGG AGA TGG G-3′, CDK6 reverse primer 5′-CAT GTG AGA CTT TGA GTA GAC CTG A-3′, GAPDH forward primer 5′-CAA GGC TGA GAA CGG GAA G-3′, and GAPDH reverse primer 5′-TGA AGA CGC CAG TGG ACT C-3′.

### Cell transfection

miR-1296-5p mimics (miR-1296-5p-mimics), negative control mimics (NC-mimics), miR-1296-5p inhibitors (miR-1296-5p-inhibitors), and negative control inhibitors (NC-inhibitors) were chemically synthesized by RiboBio (Guangzhou, China). The GC cell lines were seeded in six-well plates and cultured to 70% confluence and then transiently transfected with the indicated RNAs using Lipofectamine 2000 reagent (Invitrogen, Carlsbad, CA) in serum-free OPTI-MEM (Invitrogen, Carlsbad, CA) according to the manufacturer’s instructions. At 48 h post-transfection, the cells were harvested for real-time PCR and Western blotting.

### MTT assay

Cell viability was determined using an MTT assay. Cells (1 × 10^4^ cells/well) were seeded into 96-well plates and transfected with the indicated RNAs and cultured for 0, 24, 48, and 72 h. Ten microliters of MTT solution (10 mg/ml) was added to each well and incubated at 37°C for 3 h. The supernatant was removed and 150 μl DMSO was added for 15–20 min. The absorbance was measured using a microplate reader (Titertek Multiskan; Flow Laboratories, North Ryde, Australia) at a wavelength of 490 nm. All experiments were performed in triplicate.

### Cell migration and invasion assay

Cell migration was examined using wound healing assays and Transwell migration assay. The transfected cells were grown to 90% confluence in six-well plates. Next, a wound was created using a sterile 100 μl pipette tip. Then, the cells were further cultured in medium with 5% serum. Images were acquired using an inverted microscope (Olympus, Tokyo, Japan) after incubation for 0–48 h. In the transwell cell migration assay, the transfected cells were added on top of the transwell membrane in the upper chamber in medium without FBS, and 600 µl of medium with 10% FBS was added to the lower chamber. Cells were allowed to migrate for 24 h. Non-migrated cells were removed from the upper surface of each Transwell with a cotton swab. The inserts were washed with PBS, fixed with 100% methanol and then stained with Wright Giemsa stains and imaged. The number of cells migrating through the membrane to the lower surface was counted in five fields under a light microscope. Cell invasion was examined using a Matrigel invasion assay. The transfected cells were plated onto Transwell chambers precoated with 20 μg Matrigel (BD Biosciences) in medium without FBS in the top portion of the chamber, and medium with 10% FBS in the lower chamber served as a chemoattractant. The chambers were incubated at 37°C with 5% CO_2_, and the cells were allowed to invade for 24 h. After the fixation and staining procedures, the number of invasive cells could be quantified by counting under a microscope.

### Cell cycle analysis by flow cytometry

Cells were trypsinized, resuspended in PBS, fixed in 70% ethanol and then stained with 40 μg/ml propidium iodide (BD Pharmingen, San Jose, CA, U.S.A.) in PBS for 30 min at 37°C. Finally, the cell cycle was analyzed with a BD Biosciences FACSCalibur flow cytometer.

### Plasmid construction and dual-luciferase activity assay

The fragments of the 3′-UTR of CDK6 and EGFR mRNA, containing wild-type (wt) or mutated (mut) putative miR-1296-5p binding sites, were synthesized and inserted between the XhoI-PmeI restriction sites in a psiCHECK-2 vector (C8021; Promega, Madison, WI). Primer sequences for the full-length 3′-UTR of CDK6 mRNA (5′-GCG ATC GCT CGA GAC GAC GGC TGG ATG TAC G-3′ and 5′-CTA CGT TTA AAC TTG AGT CAC CAG GAG AAA GAT TC-3′) and EGFR mRNA (5′-ATC GCT CGA GAC AGA TGG GCA CCA ACC GCG-3′ and 5′-CTC TAG GTT TAA ACA TCT TCC TCA AGC CCC AGA C-3′) were designed. SGC-7901 and MGC-803 cells were seeded onto 24-well plates (1.5 × 10^5^ cells/well) 24 h before transfection. The recombinants in combination with the miR-1296-5p-mimics or NC-mimics were transfected with Lipofectamine™ 2000 (Invitrogen) according to the manufacturer’s instructions. The luciferase activity was analyzed using the manufacturer’s instructions. The activities of firefly and Renilla luciferases in cell lysates were determined with a dual-luciferase assay system (E1910; Promega). The Renilla luciferase activity was normalized to firefly luciferase activity for each transfected well. Three independent experiments were performed in triplicate.

### Rescue experiments

The plasmids encoding CDK6 and EGFR were purchased from YouBio Company (YouBio, Chang Sha, China). Briefly, cDNAs encoding full-length human CDK6 and EGFR were amplified from GES-1 human cDNAs and cloned into BamHI/XhoI restriction sites of the pcDNA3.1-3xFlag-C plasmid vector. Primer sequences for amplifying CDK6 (upper 5′- CGG GAT CCC GAT GGA GAA GGA C -3′ and lower 5′- CCG CTC GAG CGG TCA GGC TGT ATT -3′) and EGFR (upper 5′- CGG GAT CCC GAT GCG ACC CTC C -3′ and lower 5′- CCG CTC GAG CGG TCA CTG TGT CTG -3′) were designed. The clones were verified by sequencing. For rescue experiments, the GC cell lines were cotransfected with NC-mimics or miR-1296-5p-mimics together with pcDNA-Flag-CDK6 or pcDNA-Flag-EGFR. At 24 h post-transfection, the cells were prepared for Western blotting, MTT, and Matrigel invasion assays.

### Western blot analysis

Frozen tissues and harvested cells were washed and homogenized on ice with RAPI buffer (50 mM TRIS pH 7.5, 150 mM NaCl, 1% NP-40, 0.5% sodium deoxycholate and 1 mM phenylmethanesulfonyl fluoride). Protein concentrations were evaluated using BCA assays. Equal amounts of protein were subjected to 10% SDS-PAGE, and electroblotted to PVDF membranes (Millipore). Membranes were blocked with 5% milk for 2 h at room temperature and incubated at 4°C overnight with an anti-human EGFR rabbit polyclonal antibody (1:1000; CST, 4267), an anti-human CDK6 rabbit polyclonal antibody (1:1500; Proteintech, 14052-1-AP), an anti-human NCL rabbit polyclonal antibody (1:1500; Santa Cruz Biotechnology, sc-17826), and an anti-human GAPDH rabbit polyclonal antibody (1:5000; CST, 5174S). Then, the membranes were washed and incubated with fluorochrome-labeled secondary anti-rabbit IgG (IRDye 800-LI-COR, Odyssey) for 1.5 h at room temperature. After washing three times with TBS-T for 10 min per wash, the membranes were imaged with an Odyssey infrared imaging system (U.S.A., LI-COR). The levels of protein expression were calculated as the ratio of the intensity of protein to that of GAPDH. Experiments were conducted in triplicate.

### Immunohistochemical staining

Immunohistochemical staining was performed on formalin-fixed, paraffin-embedded GC and adjacent normal tissues. A standard immunohistochemical technique was applied. Antigen retrieval was achieved by boiling the specimens in a citrate buffer at 120°C for 3 min. After blocking endogenous peroxides and proteins, the specimens were incubated with anti-human EGFR rabbit polyclonal IgG (1:200; Proteintech; 10515-1-AP) and anti-human CDK6 rabbit polyclonal IgG (1:50; Proteintech; 15508-1-AP) antibodies overnight. After washing with PBS, sections were incubated with the secondary antibody conjugated to streptavidin-horseradish peroxidase followed by diaminobenzidine and then counterstained with hematoxylin.

### Statistical analysis

The relationship between the miR-1296-5p expression level and clinicopathologic parameters was analyzed by the chi square test. The difference between two groups was analyzed by Student’s *t*-test. For difference among multiple groups, one-way ANOVA was performed. The correlation between miR-1296-5P and EGFR or CDK62 expression was assessed by Pearson’s correlation analysis. All statistical analyses were performed using SPSS version 13.0 software (SPSS Inc., Chicago, IL, U.S.A.), and *P*<0.05 was considered statistically significant.

## Results

### miR-1296-5p is down-regulated in GC

**Figure 1:**
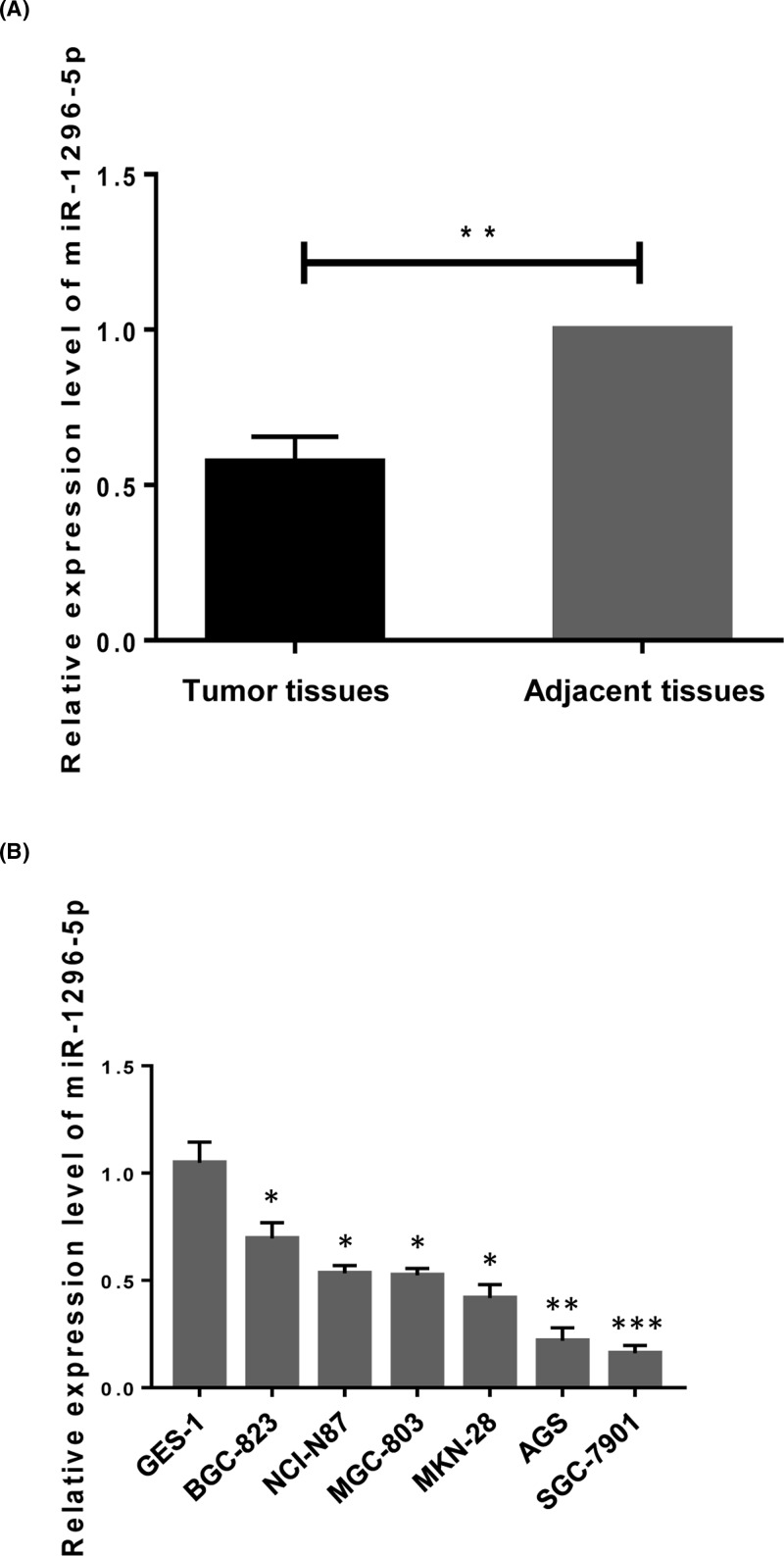
Relative expression level of miR-1296-5p miR-1296-5p is down-regulated in GC. (**A**) The expression pattern of miR-1296-5p in GC and matched adjacent normal tissues. **P<0.01 compared with the matched normal tissues. (**B**) The expression of miR-1296-5p in GES-1 normal gastric epithelial cells and six GC cell lines. Values represent the mean ± SEM. *P<0.05, **P<0.01, and ***P<0.001 vs. GES-1 cells.

First, the expression of miR-1296-5p was detected in GC tissues and their matched adjacent non-tumor tissues in 40 patients by real-time PCR. We found that miR-1296-5p expression was down-regulated in 62.5% (25/40) (*P*<0.05) of GC tissues compared to that in the corresponding adjacent normal tissues (Table 1). Quantitative analysis showed that the average expression level of miR-1296-5p was significantly lower in the cancer tissues compared with that in the non-tumor tissues (Figure 1A). Then, we measured miR-1296-5p expression in six GC cell lines, including BGC-823, NCI-N87, MGC-803, MKN-28, AGS, SGC-7901 and one normal gastric epithelial cell line, GES-1. We found that miR-1296-5p expression was notably down-regulated in the six tested GC cell lines compared with that in GES-1 cells (Figure 1B). These concordant data clearly showed that miR-1296-5p expression was down-regulated in GC.

**Table 1:** Correlation of the expression patterns of miR-1296-5p with clinicopathological features in patients with GC

Clinicopathological parameters	miR-1296-5p expression			
	Under expression (*n*=25)	Over expression (*n*=15)	X^2^	*P*-value
**Age (years)**				
<60	13	10	0.836	0.361
≥60	12	5	–	–
**Gender**				
Male	11	7	0.027	0.870
Female	14	8	–	–
**Histological grade**				
Well and moderate	13	5	1.338	0.247
Poor and not	12	10	–	–
**T STAGE**				
T1–T2	6	10	7.192	0.007
T3–T4	19	5	–	–
**TNM Stage**				
I+II	5	10	8.810	0.003
III+IV	20	5	–	–

### Decreased miR-1296-5p expression is correlated with the advanced clinical stage

We further investigated the association of miR-1296-5p expression with the clinicopathological characteristics of patients. According to the expression levels of miR-1296-5p, the clinical cases were divided into two groups based on a twofold cut-off (0.5 relative expression ratio), an underexpression group (*n*=25) and an overexpression group (*n*=15). The expression of miR-1296-5p was associated with TNM stage (χ^2^ = 8.810, *P*=0.003) but was not correlated with gender, age, or histological grade (Table 1).

### miR-1296-5p suppresses the proliferation of GC cells

**Figure 2:**
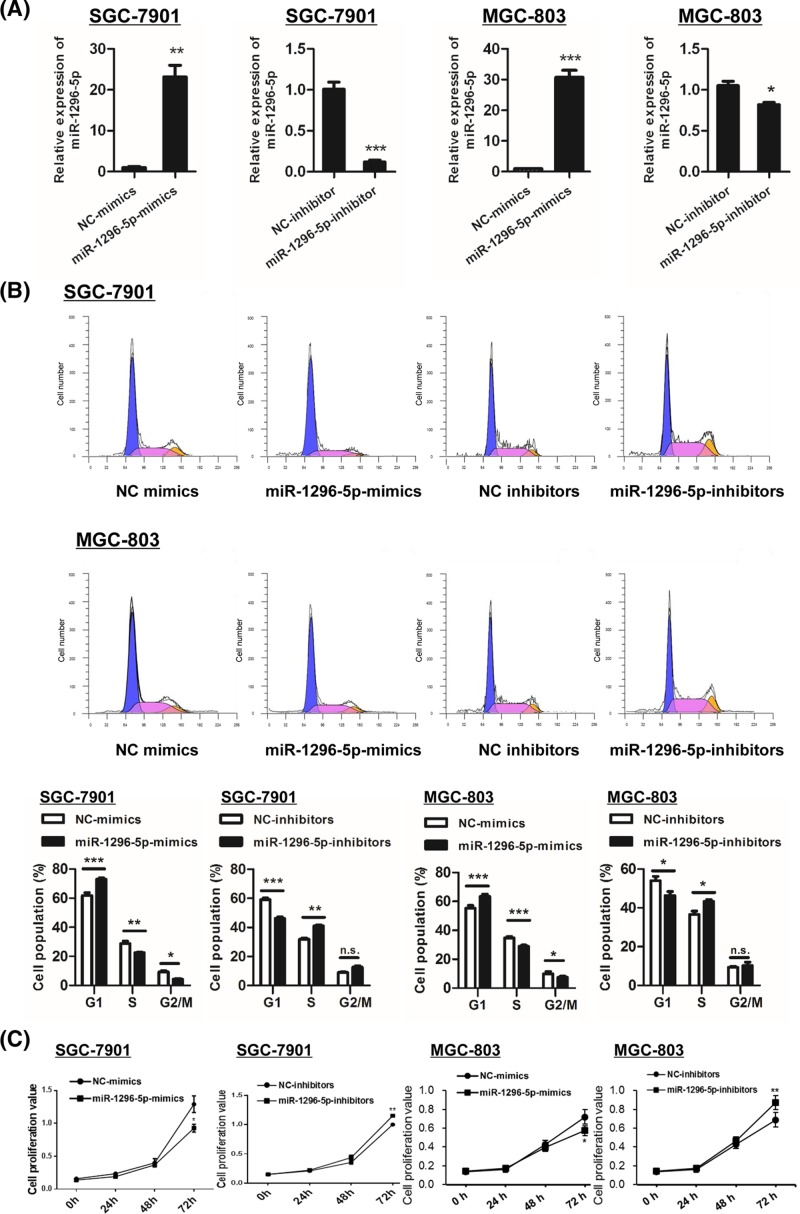
miR-1296-5p suppresses proliferation in GC cells (**A**) Quantitative RT-PCR analysis of miR-1296-5p in SGC-7901 and GSC-803 cells. (**B**) Cell cycle distribution detected by flow cytometry in SGC-7901 and GSC-803 cells. (**C**) Cell viability was examined by an MTT assay in SGC-7901 and GSC-803 cells. Bar graphs represent the mean ± SEM from at least three independent experiments; *P<0.05, **P<0.01, and ***P<0.001 vs. the indicated groups.

To evaluate the role of miR-1296-5p in cell proliferation, miR-1296-5p was overexpressed by transfection with miR-1296-5p mimics (miR-1296-5p-mimics) in SGC-7901 and MGC-803GC cell lines followed by measurements of cell cycle distribution by flow cytometry. The miR-1296-5p expression level was significantly higher in cells transfected with miR-1296-5p-mimics than that in cells transfected with NC-mimics (Figure 2A). The expression of miR-1296-5p was suppressed by transfection with antisense oligonucleotides (miR-1296-5p-inhibitors) in SGC-7901 and MGC-803 cells (Figure 2A). The proportion of cells in the S phase was decreased by transfection with miR-1296-5p-mimics in both cell lines (Figure 2B). Conversely, inhibition of miR-1296-5p resulted in an increase in the cell proportion in the S phase in the two GC cell lines (Figure 2B). We further detected cell viability using an MTT assay in the two GC cell lines. In agreement with the results observed by flow cytometry, the cell viability was decreased in miR-1296-5p-mimics cells compared with the controls, whereas transfection with miR-1296-5p-inhibitors caused an opposite effect (Figure 2C). These findings indicated a growth inhibitory role of miR-1296-5p in GC.

### miR-1296-5p suppresses the migration and invasion of GC cells.

**Figure 3:**
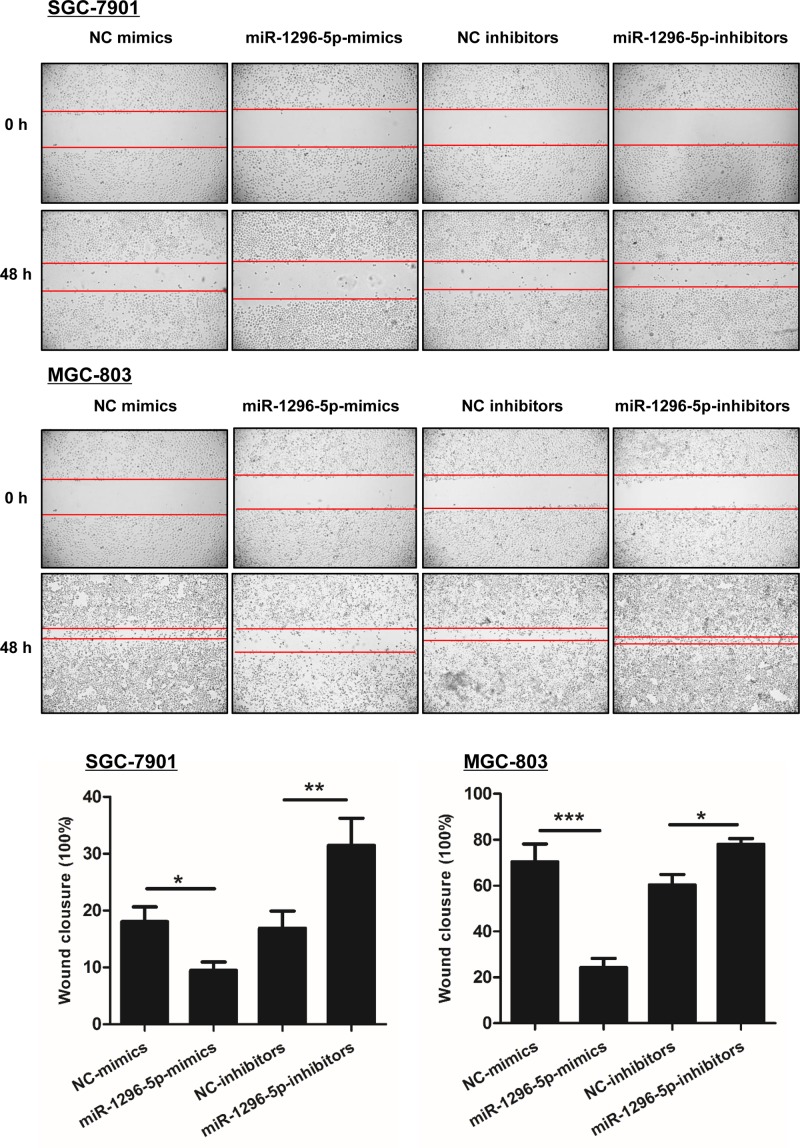
miR-1296-5p inhibits the migration of GC cells Representative results of the scratch healing assay in SGC-7901 and GSC-803 cells. Bar graphs represent the mean ± SEM from at least three independent experiments; *P<0.05, **P<0.01, and ***P<0.001 vs. the indicated groups.

**Figure 4:**
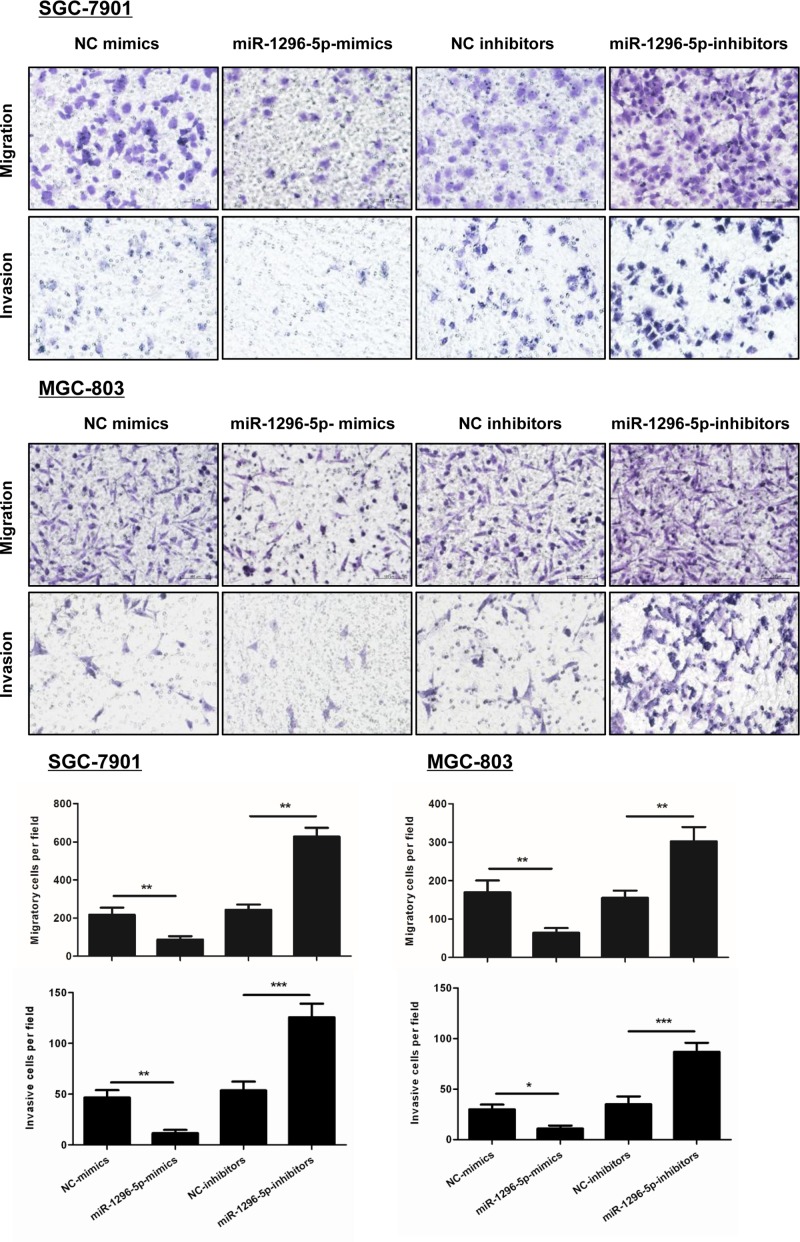
miR-1296-5p suppresses the invasion of GC cells Representative images of invasive cells on polycarbonate Transwell membranes. Bar graphs represent the average number of migratory or invasive cells from three independent experiments; *P<0.05, **P<0.01, and ***P<0.001 vs. the indicated groups.

A monolayer scratch healing assay, Transwell migration assay, and Matrigel invasion assay were performed to investigate the effect of miR-1296-5p on migration and invasion in GC cells. Overexpression of miR-1296-5p markedly inhibited cell migration in SGC-7901 and MGC-803 cells, because the percentage of wound closure was reduced in the cells transfected with miR-1296-5p-mimics compared with NC-mimics control cells (Figure 3). Furthermore, miR-1296-5p-inhibitors-transfected GC group showed a greater percentage of wound closure compared with the control group (Figure 3). As shown by the Transwell migration and Matrigel invasion assay, forced expression of miR-1296-5p significantly impaired the migration and invasion of both SGC-7901 and MGC-803 cells, while the number of cells that migrated and invaded through the microporous membrane was increased by transfection with miR-1296-5p-inhibitors (Figure 4), which suggested that miR-1296-5p suppresses the migration and invasion of GC cells.

### Identification of miR-1296-5p target genes

**Figure 5:**
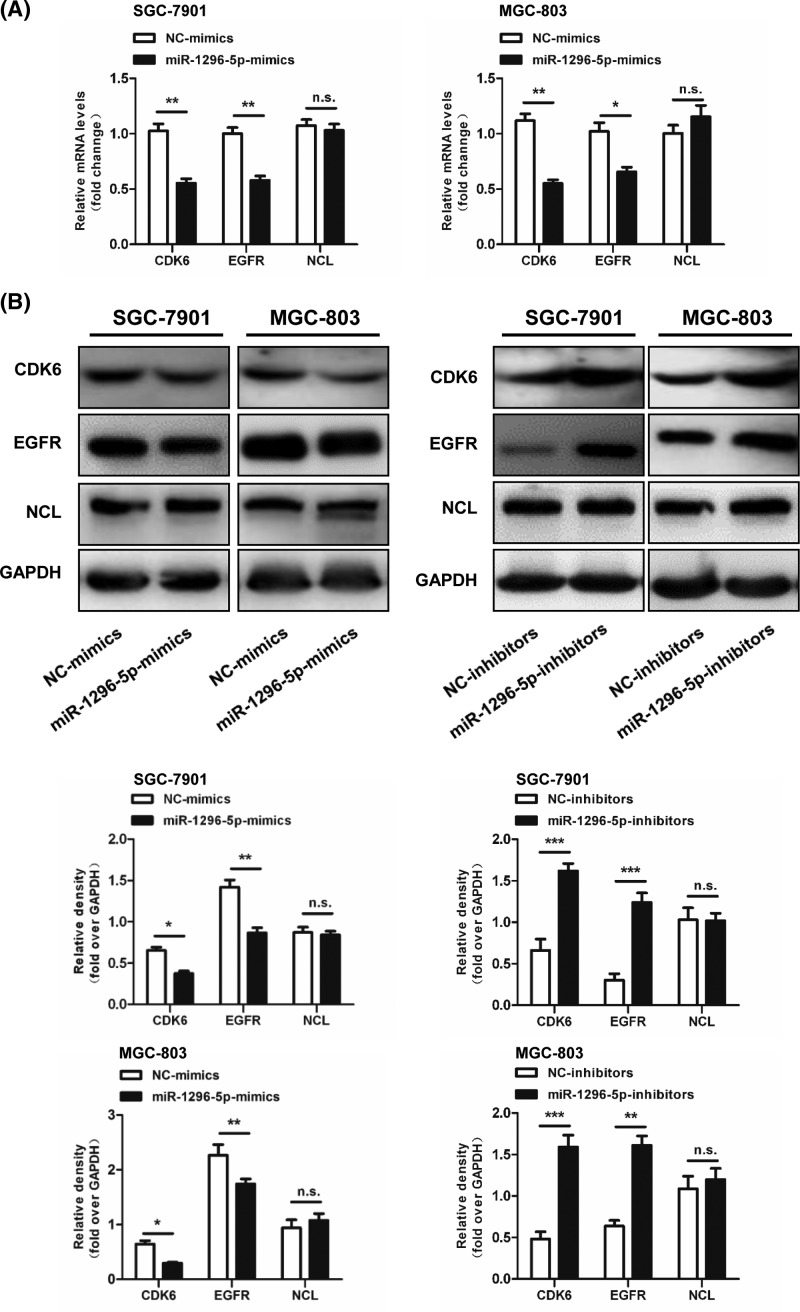
Identification of miR-1296-5p target genes (**A**) Relative mRNA levels of CDK6, EGFR, and NCL in SGC-7901 and GSC-803 cells. (**B**) Representative Western blots for CDK6, EGFR, and NCL in SGC-7901 and GSC-803 cells. Bar graphs represent the mean ± SEM from three independent experiments; n.s. indicates not statistically significant; *P<0.05, **P<0.01, and ***P<0.001 vs. the indicated groups.

Because we found that miR-1296-5p played an inhibitory role in proliferation and migration in GC, we aimed to search for the target genes of miR-1296-5p related to tumorigenesis. There are three putative targets predicted by online search tools (e.g., TargetScan. Microrna.org and PicTar), including CDK6, EGFR, and NCL. To identify the target genes of miR-1296-5p in the three predicted targets, we detected the mRNA and protein expression levels of these three candidate genes. Compared with the control cells transfected with NC-mimics, overexpression of miR-1296-5p in SGC-7901 and MGC-803 cells suppressed mRNA and protein expression of both CDK6 and EGFR, but not NCL (Figure 5A,B). The protein expression levels of CDK6 and EGFR were elevated by inhibition of miR-1296-5p in SGC-7901 and MGC-803 cells; however, the expression of NCL was not affected (Figure 5B). These results suggested that the target genes of miR-1296-5p might be CDK6 and EGFR.

### EGFR and CDK6 are targets of miR-1296-5p

**Figure 6:**
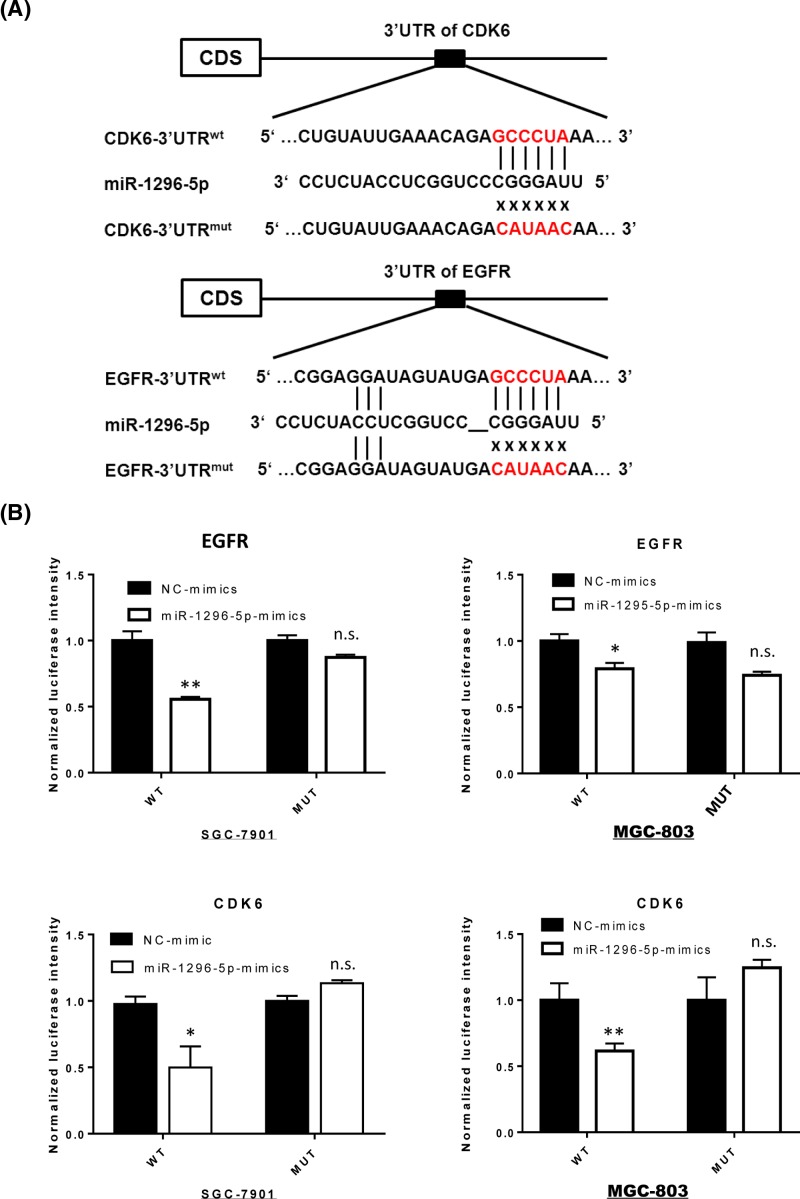
CDK6 and EGFR are targets of miR-1296-5p (**A**) Schematic graph of wild-type and mutant putative binding sites of miR-1296-5p targeting the 3′-UTR of CDK6 and EGFR. (**B**) Relative luciferase activity of SGC-7901 and GSC-803 cells after cotransfection with wild-type or mutant CDK6 and EGFR 3′-UTR reporter genes and miR-1296-5p-mimics. Bar graphs represent the mean ± SEM; n.s. indicates not statistically significant; *P<0.05 and **P<0.01 vs. NC-mimics.

The putative binding sites of miR-1296-5p in the 3′-UTR of EGFR and CDK6 were predicted using TargetScan prediction software (Figure 6A). To investigate whether EGFR and CDK6 were direct targets of miR-1296-5p, we constructed luciferase reporter vectors with the 3′-UTR regions of CDK6 and EGFR mRNA, which contain wild-type or mutant miR-1296-5p binding sequences. The luciferase reporter vectors were cotransfected with miR-1296-5p-mimics or NC-mimics into SGC-7901 and MGC-803 cells. In both GC cell lines, the relative luciferase activity of psiCHECK-2/CDK6-3′-UTR^wt^, but not psiCHECK-2/CDK6-3′-UTR^mut^, was significantly suppressed by transfection with miR-1296-5p-mimics compared with that of NC-mimics (Figure 6B). Furthermore, miR-1296-5p-mimics also down-regulated the luciferase activity of the psiCHECK-2/EGFR-3′UTR^wt^ compared with that in the NC group. Overall, these results indicated that CDK6 and EGFR were the direct target genes of miR-1296-5p.

**Figure 7:**
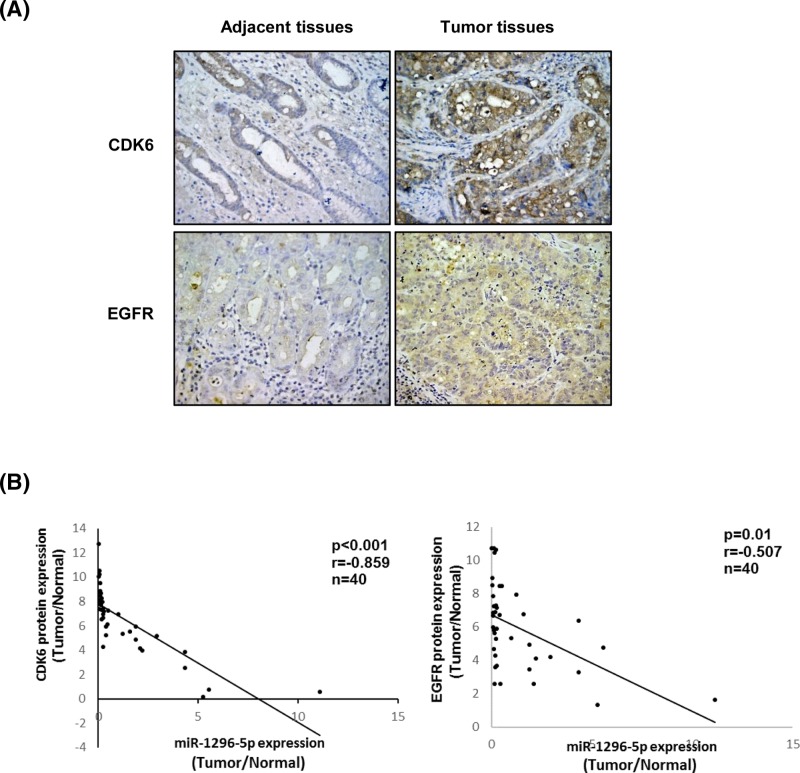
EGFR and CDK6 are down-regulated in GC and negatively correlated with miR-1296-5p (**A**) Representative images from immunohistochemical staining of CDK6 and EGFR in GC and matched adjacent normal tissues. (**B**) Correlation between miR-1296-5p expression levels and CDK6 and EGFR protein expression levels in paired normal/tumor GC tissue samples.

The expression levels of CDK6 and EGFR were further observed in GC and their matched adjacent non-tumor tissues of 40 patients by immunohistochemical staining followed by the correlation analysis of the expression levels of the two proteins and miR-1296-5p. The protein expression levels of both CDK6 and EGFR were greater in GC tissues than those in adjacent normal tissues (Figure 7A). Pearson’s correlation analysis showed that the expression levels of CDK6 and EGFR were both inversely correlated with the expression of miR-1296-5p (*r* = −0.859, *P*<0.001 and *r* = −0.507, *P*=0.01, respectively) in GC tissues (Figure 7B).

### Downregulation of EGFR and CDK6 is key to the tumor-suppressive function of miR-1296-5p

**Figure 8:**
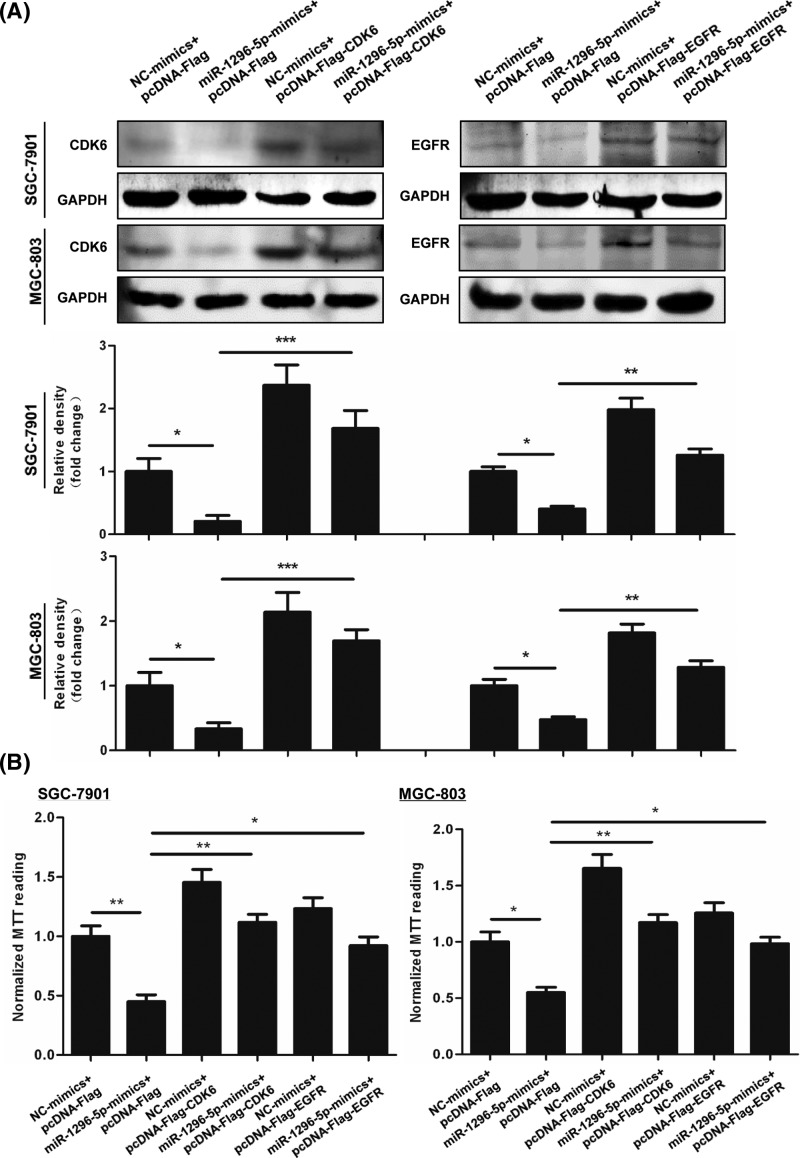
Downregulation of EGFR and CDK6 are key to the antiproliferative function of miR-1296-5p (**A**) Representative Western blots for CDK6 and EGFR in SGC-7901 and GSC-803 cells. (**B**) Cell viability was examined by an MTT assay in SGC-7901 and GSC-803 cells. Bar graphs represent the mean ± SEM from at least three independent experiments; *P<0.05, **P<0.01, and ***P<0.001 vs. the indicated groups.

**Figure 9:**
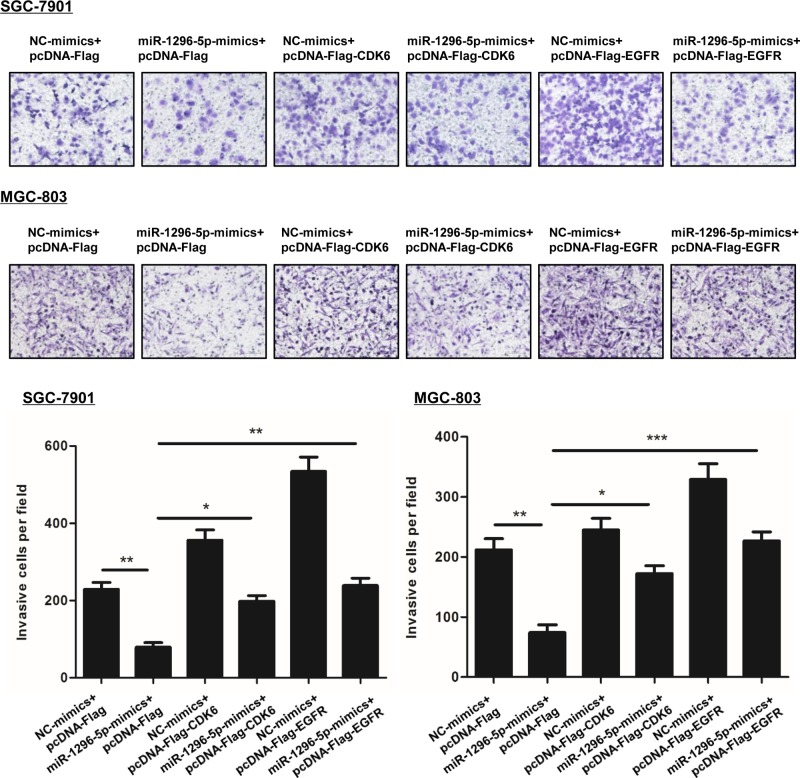
Overexpression of EGFR and CDK6 reversed the inhibitory effect of miR-1296-5p on cell invasion Representative images of invasive cells on polycarbonate Transwell membranes. Bar graphs represent the average number of invasive cells per field from three independent experiments; *P<0.05, **P<0.01, and ***P<0.001 vs. the indicated groups.

Since miR-1296-5p targets CDK6 and EGFR, we tested the effect of CDK6 and EGFR on the inhibition of proliferation and invasion that was observed by overexpressing miR-1296-5p. Transfection with the vectors expressing Flag-tagged CDK6 and EGFR significantly increased the protein expression levels of CDK6 and EGFR (Figure 8A). Transfection of SGC-7901 and MGC-803 cells with miR-1296-5p-mimics decreased cell proliferation, which was reversed by overexpressing CDK6 or EGFR. Then, we measured cell invasion by a Matrigel invasion assay. Overexpression of CDK6 and EGFR reversed the inhibited invasion caused by miR-1296-5p-mimics (Figure 9).

## Discussion

The present study focused on the role of miR-1296-5p in the process of gastric carcinogenesis and development. We found that miR-1296-5p was down-regulated in GC tissues and cell lines, and the decreased expression level of miR-1296-5p was clinically associated with the advanced clinical stage. In the typical gastric cell lines SGC-7901 and MGC-803, examination of miRNA function by overexpression and inhibition showed that miR-1296-5p negatively regulated cell proliferation, migration, and invasion. miRNAs function by regulating the expression of their target genes. In the present study, we identified CDK6 and EGFR as novel targets of miR-1296-5p. Overall, our results indicate a tumor-suppressive role of miR-1296-5p through the translational repression of oncogenic CDK6 and EGFR in GC.

miRNAs are a class of non-coding RNA molecules, 19–24 nucleotides in length, that negatively regulate gene expression by base-pairing with complementary sequences in the 3′-UTR of targeted mRNAs [8]. miRNAs have been reported to be aberrantly regulated during carcinogenesis and have a wide range of abilities to regulate cell survival, proliferation, differentiation, migration, invasion, and metastasis in a variety of cancerous tumors [23]. miR-1296-5p, a novel cancer-related miRNA, has been found to be dysregulated in various cancers. miR-1296-5p acts as a tumor suppressor in reproductive system tumors, which has been reported to decrease the proportion of cells in the S phase in prostate cancers [15]. Liu et al. found that miR-1296-5p also induces cell apoptosis by targeting the PIM1-STAT3 pathway in cervical cancer [16]. miR-1296 suppresses cell growth in triple-negative breast cancer cell lines and plays a potential role in sensitizing breast cancer cells to cisplatin [17]. Moreover, miR-1296 has been shown to increase chemoresistance and could be used as a new potential biomarker for breast cancer stem cells [24]. It has been reported that miR-1296 also plays an important role in digestive system tumors. miRNA-1296 inhibits metastasis and the epithelial-mesenchymal transition of hepatocellular carcinoma cells by targeting the SRPK1-mediated PI3K/AKT pathway [19]. Zhu et al. showed that miR-1296 regulated cell migration and invasion in human GC by targeting the ERBB2/Rac1 signaling pathway [18]. Consistent with previous studies, our results also support an inhibitory role of miR-1296 in tumorigenesis and progression of GC. Each human miRNA regulates the expression of several targets [25]. miR-1296-5p targets different genes and cancer pathways in different contexts as described above; therefore, we identified two novel targets, CDK6 and EGFR, for miR-1296-5p to provide an understanding of the underlying mechanism.

CDK6, a D-type cyclin-dependent kinase, propels cell cycle progression from the G1 to the S phase through the phosphorylation and subsequent inactivation of the retinoblastoma 1 (RB1) protein [26]. CDK6 has been shown to be a tumor promoter in many types of cancers. The expression level of CDK6 is elevated in pancreatic cancer, medulloblastoma, and GC [27–29]. Furthermore, overexpression of CDK6 is correlated with poor prognosis and might be a molecular marker for the prognostic assessment of medulloblastoma [30]. CDK6 has been shown to be regulated by a series of miRNAs, including miR-29, miR-129, miR-107, miR-22, and miR-195 [29–33]. In the present study, we found that CDK6 was overexpressed in GC tissues compared with paired normal tissues, which was negatively correlated with the expression of miR-1296-5p. Overexpression of miR-1296-5p mimics inhibited the expression of CDK6 in GC cells, and we further confirmed CDK6 and EGFR as targets of miR-1296-5p using luciferase reporter assays.

EGFR is a cell membrane tyrosine kinase receptor belonging to the ErbB family. High levels of EGFR are observed in various cancers, including non-small cell lung cancer, colorectal cancer, pancreatic cancer, esophagogastric cancer, and GC [34–38]. Overexpression of EGFR is associated with an increased risk of invasion or metastasis, while the inhibition of EGFR leads to decreased cancer cell division, migration, angiogenesis, and apoptosis in solid tumors [39]. It has been reported that EGFR is the target of several miRNAs, including miR-34a, miR-133a, and miR-491-5 [40–42]. In the present study, we identified EGFR as the target of miR-1296-5p. The expression of tumor promoters is regulated by several miRNAs, and one miRNA may regulate the expression of several target genes, which form a regulatory network in tumorigenesis and cancer progression. Our findings provide some limited details of the complex networks.

CDK6, an oncogene, promotes cell proliferation in many kinds of cancers and regulates cell migration [43]. EGFR contributes to cancer progression by mediating cellular proliferation, migration, adhesion and metastasis. The overexpression of CDK6 and EGFR reversed the inhibited invasion caused by miR-1296-5p-mimics. miR-1296-5p acts as a tumor suppressor, at least partly, by targeting CDK6 and EGFR.

In summary, the present study showed that miR-1296-5p inhibited cell proliferation, migration, and invasion in GC by targeting CDK6 and EGFR. The present study should provide new insights into the molecular basis underlying the metastasis of GC, suggesting that the modulation of miR-1296-5p suppresses GC.

## Abbreviations

AJCC: American Joint Committee on Cancer
CDK6: Cyclin-dependent kinase 6
cDNA: complementary DNA
EGFR: epidermal growth factor receptor
FBS: fetal bovine serum
GC: Gastric cancer
mut: mutated
TNM: tumor-node-metastasis
wt: wild-type
